# Study of cabbage antioxidant system response on early infection stage of *Xanthomonas campestris* pv. *campestris*

**DOI:** 10.1186/s12870-024-04994-w

**Published:** 2024-04-24

**Authors:** Zeci Liu, Jie Wang, Zhibin Yue, Jue Wang, Tingting Dou, Tongyan Chen, Jinbao Li, Haojie Dai, Jihua Yu

**Affiliations:** College of Horticulture, Gansu Agriculture University, Lanzhou, 730070 People’s Republic of China

**Keywords:** *Brassica oleracea*, Black rot, Antioxidant system, Omics, Resistance mechanism

## Abstract

Black rot, caused by *Xanthomonas campestris* pv. *campestris* (*Xcc*) significantly affects the production of cabbage and other cruciferous vegetables. Plant antioxidant system plays an important role in pathogen invasion and is one of the main mechanisms underlying resistance to biological stress. Therefore, it is important to study the resistance mechanisms of the cabbage antioxidant system during the early stages of *Xcc*. In this study, 10^8^ CFU/mL (OD_600_ = 0.1) *Xcc* race1 was inoculated on “zhonggan 11” cabbage using the spraying method. The effects of *Xcc* infection on the antioxidant system before and after *Xcc* inoculation (0, 1, 3, and 5 d) were studied by physiological indexes determination, transcriptome and metabolome analyses. We concluded that early *Xcc* infection can destroy the balance of the active oxygen metabolism system, increase the generation of free radicals, and decrease the scavenging ability, leading to membrane lipid peroxidation, resulting in the destruction of the biofilm system and metabolic disorders. In response to *Xcc* infection, cabbage clears a series of reactive oxygen species (ROS) produced during *Xcc* infection via various antioxidant pathways. The activities of antioxidant enzymes such as superoxide dismutase (SOD), peroxidase (POD), and catalase (CAT) increased after *Xcc* infection, and the ROS scavenging rate increased. The biosynthesis of non-obligate antioxidants, such as ascorbic acid (AsA) and glutathione (GSH), is also enhanced after *Xcc* infection. Moreover, the alkaloid and vitamin contents increased significantly after *Xcc* infection. We concluded that cabbage could resist *Xcc* invasion by maintaining the stability of the cell membrane system and improving the biosynthesis of antioxidant substances and enzymes after infection by *Xcc*. Our results provide theoretical basis and data support for subsequent research on the cruciferous vegetables resistance mechanism and breeding to *Xcc*.

## Introduction

Cabbage is a kind of vegetable with high yield, long storage life and resistant to transportation. Moreover, it is rich in many kinds of flavonoids, glucosinolates, and other biologically active ingredients and nutrients, and is loved by most consumers [1, 2]. Therefore, cabbage consumption has increased in recent years. To meet market demand, cabbage is accompanied by rapid expansion of the cultivation area and general improvement of the multiple planting index. However, cabbage are susceptible to infection by numerous fungal and bacterial pathogens, including *Xanthomonas campestris* pv. *campestris* (*Xcc*) [3–6].

Black rot (BR), caused by *Xanthomonas campestris* pv. *campestris* (*Xcc*) is one of the three major diseases of cabbage and other cruciferous vegetables [7–11]. As a seed-born disease, *Xcc* has been spread internationally through the seed trade, it also can spread by insects, wind, blowing rain, aerosols, sprinkler irrigation and farm tools. *Xcc* enters the host plants vascular system through stomata, hydathodes, wounds caused by machinery or insects. The bacteria causes systemic vascular disease, the typical symptoms are vein blackening, leaf tissue necrosis and V-shaped chlorotic lesions [12]. In recent years, BR has been prevalent in the main production areas of cabbage in the world, resulting in a serious decline in quality and yield [7, 8, 13]. Due to the lack of effective antigens, the research on the discovery and identification of BR resistance genes and the breeding of cabbage for BR resistance has been slow [8, 9, 12, 14]. Therefore, it is of great significance to study the resistance mechanisms of BR in cabbage for chemical less production and resistance breeding.

Studies have shown that infection with pathogenic bacteria can affect the physiological and biochemical reactions in plants, including photosynthetic performance, hormone content, and antioxidant systems [15–17]. The antioxidant system, which is composed of various antioxidant enzymes and antioxidants, plays an important role in plant responses and adaptation to biological stress [18, 19]. Under stress, plants gradually produce large amounts of hydrogen peroxide (H_2_O_2_), superoxide anions (O_2_•^−^), hydroxide ions (OH^−^), and hydroxyl radicals (•OH), which lead to excessive accumulation of biological reactive oxygen species (ROS) [20–22]. The production of large amounts of ROS leads to a serious imbalance in the scavenging ability of the antioxidant system, resulting in damage to plant somatic cells and other important physiological processes, leading to cell apoptosis and death [23, 24]. During evolution and development, plants developed a relatively perfect stress defense system to balance the production and removal of ROS [25, 26]. Antioxidant systems can be classified as antioxidant enzymes and non-enzymes (antioxidants). Both systems work together to limit intracellular ROS levels and protect cells from oxidative damage. Superoxide dismutase (SOD), peroxidase (POD), catalase (CAT), ascorbate peroxidase (APX), glutathione reductase (GR), glutathione peroxidase (GPX), and dehydroascorbate reductase (DHAR) are some of the common cellular enzymes that protect against ROS damage [27–30]. Glutathione (GSH) and ascorbic acid (AsA) are common plant antioxidants. In addition to AsA, cabbage contains carotenoids, polyphenols, flavonoids, anthocyanins, and other antioxidant substances [31–33]. Therefore, it is important to study the changes in the related enzymes and antioxidant substances in these antioxidant systems to prevent and control cabbage BR.

By measuring the physiological indexes of broccoli and cabbage, it was found that the activities of antioxidant enzymes such as SOD and POD decreased after *Xcc* infection [19, 34]. In addition, transcriptome and metabolomic studies have found that antioxidant genes and substances also show significant responses after *Xcc* infection [10, 35–43]. However, most of these studies have focused on using single physiological indexes assay, transcriptomics, metabonomics, proteomics or MicroRNAs analysis to analyze the mechanism of the cabbage antioxidant system response to BR infection. Some of the results of these studies were different due to different plant materials, methods of infection, and physiological species of *Xcc.*

In this study, *Xcc* was inoculated on cabbage at the seedling stage. The physiological indexes on different days in the early stage of infection were determined, and the responses of related genes and antioxidant substances were analyzed using transcriptomics and metabolomics. The experimental results will provide a reference for revealing the relationship between BR resistance and the antioxidant system of cabbage seedlings and provide a basis for further research on the mechanism of BR resistance in cabbage and breeding new varieties resistant to BR.

## Materials and methods

### Plant materials and inoculation

Cabbage seeds (“Zhonggan 11″ variety, susceptible to *Xcc*) were used as the plant material, and *Xanthomonas campestris* pv. *campestris* (*Xcc*) race 1(the most pathogenic and common physiological species in cabbage production) was selected as the pathogenic bacterium. The seeds were sown in diameter 7 cm plastic pots filled with sterilized soil and incubated in a glasshouse with a minimum temperature of 20/15℃ (day/night). When the seedlings had four leaves (approximately 40 d after sowing). *Xcc* was inoculated by spraying method, the inoculation method was referred to previous research [12]. The concentration of pathogenic *Xcc* was 10^8^ CFU/mL (OD_600_ = 0.1). After inoculation, the plants were placed in a greenhouse conditions at 25–28℃, with a relative humidity > 95%.

### Sampling time and method

Due to the long time from *Xcc* inoculation to occur leaf characteristic disease (10 ∼ 14 d), and the short time for plant antioxidant system to respond to pathogen stress, the sampling time was scheduled based on relevant references and preliminary experiments. Leaves samples at different time points before and after *Xcc* infection (0, 1, 3, and 5 d) were collected for follow-up antioxidant-related index analysis. To ensure the accuracy of the determination results, at least five seedlings with basically the same growth status were used for the follow-up of each index determination. The measurement and sampling time of all indexes were carried out at the same time on different days after *Xcc* inoculation. The leaves used for physiological, transcriptome, and metabolome analyses were collected and quickly frozen in liquid nitrogen; three replicates were taken from each time point for physiological and transcriptome determination while six replicates were taken for metabolome determination.

### Determination of antioxidant indexes

#### Determination of membrane permeability, content of malondialdehyde, and free proline

Cabbage leaves of similar age were selected, rinsed with water, and dried, and small discs were drilled using a hole punch to avoid the main leaf vein. Ten slices were selected for each treatment and placed into beakers for the corresponding treatment. The conductivity was measured directly using a DDSJ-308 A conductivity meter (Shanghai Precision, Shanghai, China). The relative permeability and damage rate of the cell plasma membrane were calculated based on the electrical conductivity measurements. Malondialdehyde content was determined using a kit (Suzhou Kming Biotechnology Co., Ltd., Suzhou), and proline (Pro) content in leaves was determined using the ninhydrin method [44].

#### Determination of soluble sugar and soluble protein

Soluble sugar content was determined using the anthrone method [45], and soluble protein content was determined using Coomassie brilliant blue staining [46].

#### Histochemical staining

Nitroblue tetrazolium (NBT) and 3,3-diaminobenzidine (DAB) staining were used to detect the accumulation of hydrogen peroxide (H_2_O_2_) and superoxide anion (O_2_^•−^), respectively [47]. The functional leaves of cabbage were taken and placed in triangular flasks containing 0.1% (w/v) NBT-10 mol potassium phosphate buffer (pH = 7.8) and 1 mg/mL DAB aqueous solution (pH = 7.0), and the leaves were completely immersed in the dyeing buffer and placed under vacuum conditions for 0.5 h and 1 h, respectively. The leaves were incubated at room temperature in the dark for 2 h and 24 h. The leaves were then boiled in an acetic acid: glycerin: ethanol (1:1:3 (v/v)) decolorizing solution to remove the chlorophyll. After cooling, the sample was transferred to a fresh decolorizing solution and photographed using an EPSON expression 11000XL color image scanner (Win RHIZO Pro LA2400, Canada).

#### Determination of reactive oxygen species content and antioxidant enzyme activities

The content of H_2_O_2_ and superoxide anion O_2_•^−^ and the activity of antioxidant enzymes SOD, POD, and CAT were determined using kits (Suzhou Keming Biotechnology Co., LTD., Suzhou) according to the manufacturer’s instructions.

### 2.3.Determination of antioxidant activity and non-enzymatic antioxidant content in AsA-GSH cycle

Reduced AsA, DHA, reduced GSH, and oxidized glutathione (GSSG) contents and GR, DHAR, monodehydroascorbate reductase (MDHAR), and APX enzyme activities in the ASA-GSH cycle were determined by kits (Suzhou Keming Biotechnology Co., LTD., Suzhou) according to the manufacturer’s instructions.

### Analysis of differentially expressed antioxidant-related genes

#### Transcriptome analysis of differentially expressed antioxidant-related genes on different days after *Xcc* infection

Differentially expressed genes (DEGs) between uninfected leaves and *Xcc*-infected leaves related to antioxidants were identified using EBSeq_DESeq2 software, with |log2 fold change (FC)| ≥1 and Pvalue < 0.05. DEGs on different infection days were clustered based on the short time-series expression miner (STEM) cluster method. Gene Ontology (GO) and Kyoto Encyclopedia of Genes and Genomes (KEGG) pathway enrichment analyses were performed using the BMK Cloud platform2 (http: international.biocloud.net).

#### Metabolomics and data analysis

Different antioxidant metabolites were screened and classified according to different metabolite expression levels using the BMK Cloud platform2. Cluster heat maps were drawn according to the metabolite expression levels using Origin 2023b.

#### qRT-PCR verification of part of antioxidant-related gene expression levels

Total RNA was isolated using the TRIzol reagent (TIANGEN, Beijing, China) according to the manufacturer’s instructions. Genomic DNA (gDNA) removal, reverse transcription, and qRT-PCR were performed using the appropriate kits (TransGen Biotech). The *GAPDH* gene was used as the internal control (Table 1). RT-PCR was performed using SYBR Green 1 (TIANGEN, Beijing, China) on a Roche LightCycler 480 system. All reactions were performed with three technical and three biological replicates. Relative gene expression was calculated using the comparative CT method [48].

**Table 1:** Primers used for the qRT-PCR

Gene	Gene ID	Primer Sequence (5′ to 3′)
*GAPDH*	Bo5g021670	F: AACCTGACCTCGTCCAGATTCTCC R: GCTTCTGTAGCTGTCGCCTTGATAG
*SOD*	Bo5g009310	F: AGTCGCAGTCTTGAACAGCAGTG R: ACCAAGAGCATGGACATGGAAACC
*POD*	Bo8g115240	F: TCCGTCGCACCACAGTTCAATATG R: ATAGCGTCTCTTTCGGCATCCTTG
*CAT*	Bo5g030530	F: TTTCTGCCCTGCTATTGTGGTTCC R: GTTTGGTCCTAGACGGTGCCTTTG
*APX*	Bo8g112810	F: GTTGGAGCCTATCAGAGAGCAGTTC R: AGGAATCTCAGGACCACCAGTAACC
*DHAR*	Bo8g068570	F: GCCGTTGGTGCTCCTGATGTTC R: CCACTTAGGTTTGTCGGAGAGGTTG
*GR*	Bo4g119790	F: GAGAGGCATTGAGTTCCACACAGAG R: CCCATCAACAGTTCCCTTGCTAGTC
*MDHAR*	Bo6g066960	F: GAGGTCACTGGAAGCCGACATTG R: ACAGCGTAAACATCAGGGACACTTG

### Statistical analysis

All results are presented as the mean of three independent replicates, and the data are expressed as the mean ± standard error (SE). Statistical analysis was performed using Duncan’s multiple range test (*p* < 0.05) using SPSS 22.0 (SPSS Institute Inc., USA).

## Results

### Effect of *Xcc* infection on the osmotic regulation system of cabbage

**Fig. 1:**
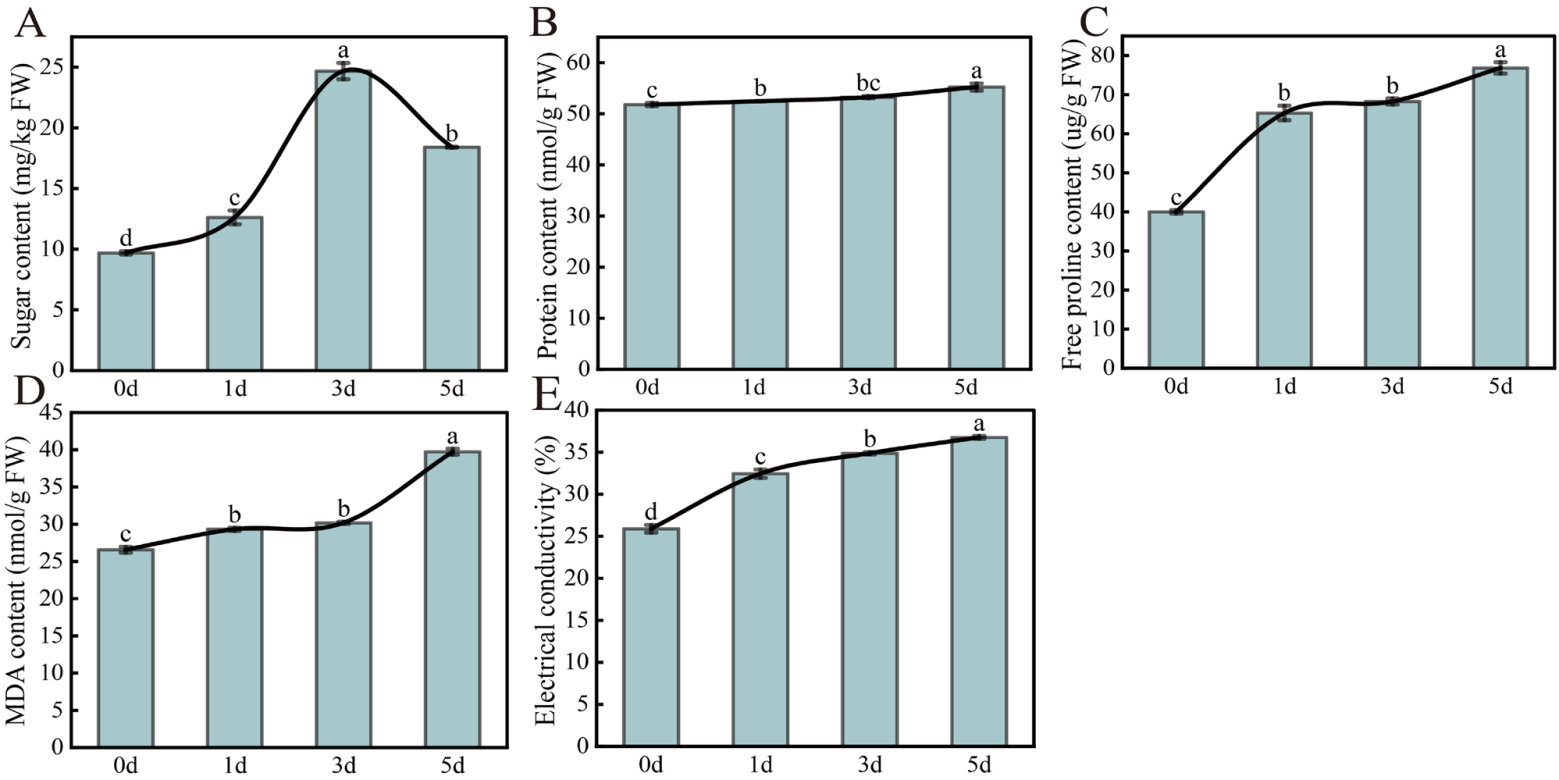
Effect of early *Xcc* infection on the osmotic regulation system. (**A**) soluble sugar; (**B**) soluble protein; (**C**) free proline; (**D**) malondialdehyde; (**E**) electrical conductivity. Mean ± standard error (SE), range, and coefficients of variation (CVs) for the antioxidant traits in the species analyzed. Species means with different letters are significantly different at *p* ≤ 0.05. Standard errors are indicated by bars. The same below

To study the effect of *Xcc* on the osmotic regulation system of cabbage, the soluble sugar, soluble protein, free proline (Pro), and malondialdehyde (MDA) contents were determined before and after *Xcc* infection. Soluble sugars, soluble proteins, and Pro are not only important osmoregulatory substances but are also important indexes that reflect plant stress resistance. The results showed that the soluble protein, Pro, and MDA contents continued to increase after *Xcc* infection from 0 to 5 d, whereas the soluble sugar content increased from 0 to 3 d and then decreased from 3 d to 5 d (Fig. 1A-D).

When plants are affected by stress, cell membrane and membrane permeability were damaged, and electrolyte extravasation, which leads to the leakage of materials inside the cell and an increase in electrical conductivity. Therefore, resistance can be determined by measuring and comparing the electrical conductivity of the cabbage before and after *Xcc* infection. Similar to the soluble sugar, soluble protein, and Pro results, electrical conductivity continued to increase after *Xcc* infection, indicating that the cell membrane was damaged and the cell extract extravasated after *Xcc* infection (Fig. 1E).

**Fig. 2:**
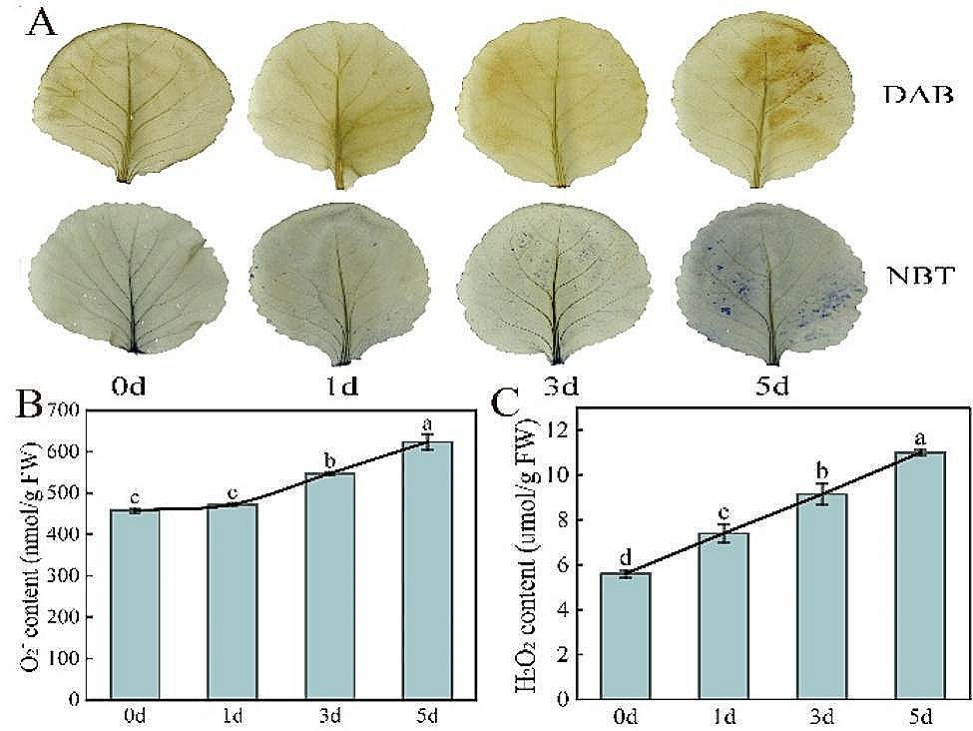
Effect of early *Xcc* infection on O_2_^−^ and H_2_O_2_ contents. (**A**) DAB and NBT staining; (**B**) O_2_^•−^ content; (**C**) H_2_O_2_ content

DAB and NBT staining could intuitively reflect the accumulation of hydrogen peroxide (H_2_O_2_) and superoxide anion (O_2_^•−^). Fig. 2A shows that the color of DAB and NBT staining was more intense with an increase in the number of *Xcc* inoculation days. The results of H_2_O_2_ and O_2_^•−^ also showed that the contents of H_2_O_2_ and O_2_^•−^ both increased continuously after *Xcc* inoculation (Fig. 2B, C). Compared with 0 d, the H_2_O_2_ and O_2_^•−^ contents on 5 d increased by 96.4% and 36.1%, respectively.

### Effect of *Xcc* infection on cabbage SOD, POD, and CAT enzymes activities and related genes expression levels

**Fig. 3:**
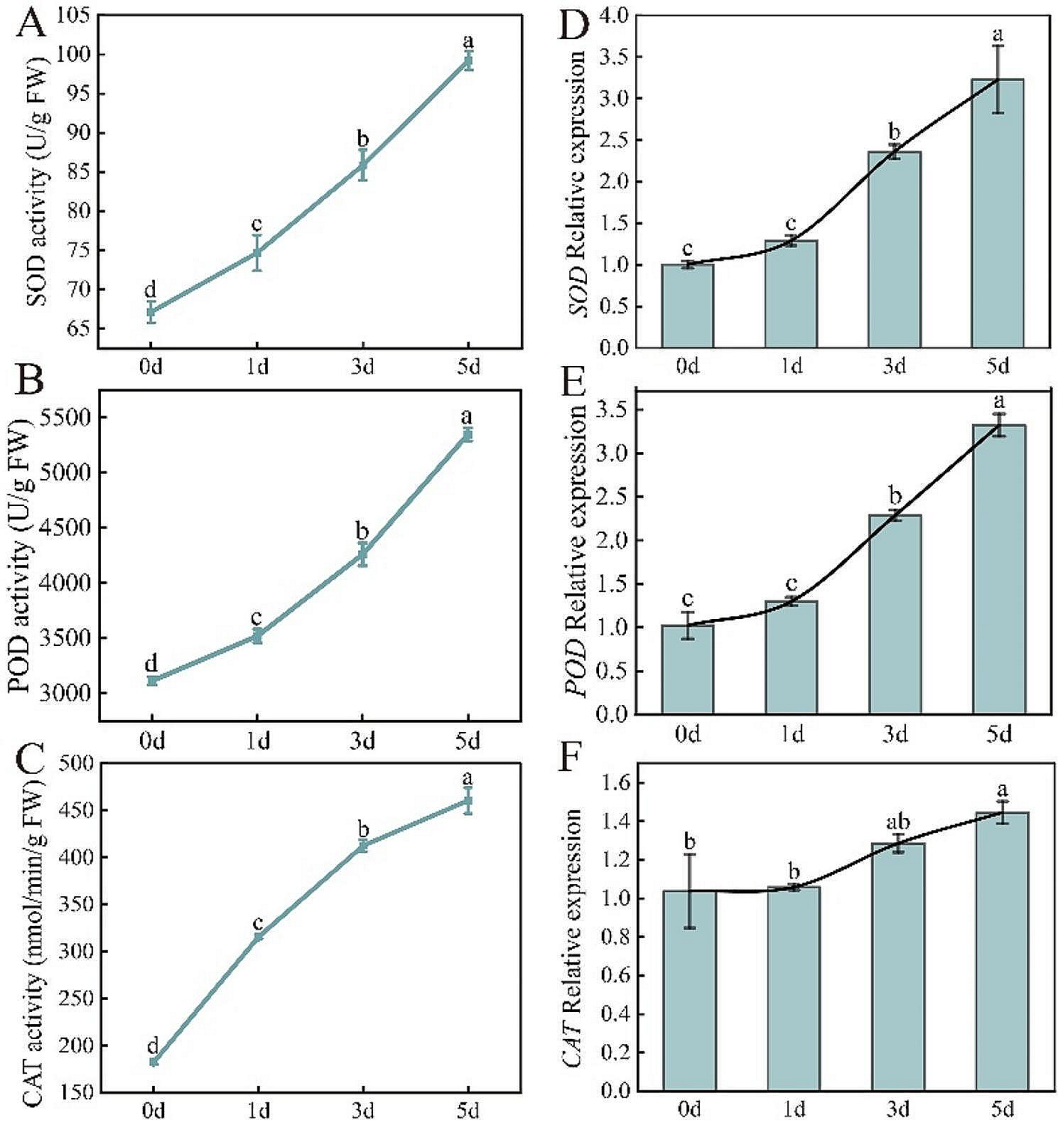
Effect of early *Xcc* infection on SOD, POD, and CAT enzymes. (**A**) SOD activity; (**B**) POD activity; (**C**) CAT activity; (**D**) *SOD* gene expression level; (**E**) POD gene expression level; (**F**) *CAT* gene expression level

Fig. 3A-C shows that SOD, POD, and CAT enzyme activities increased after *Xcc* infection. Compared to 0 d, the activities of SOD, POD, and CAT after 5 d of infection increased by 47.8%, 71.3%, and 152.3%, respectively. Similar to the activity determination results of the three antioxidant enzymes, the expression of genes (*SOD*, *POD*, and *CAT*) controlling the biosynthesis of the three enzymes also increased significantly after *Xcc* infection. In addition, the expression levels of these three genes showed a slow increase from 0 to 1 day after infection and a significant increase from 1 to 5 days after infection (Fig. 3D-F). This might be related to the rapid propagation of *Xcc* in plants after infection, resulting in leaf damage and increased ROS accumulation.

### Effect of *Xcc* infection on cabbage AsA-GSH cycle

#### Effect of ***Xcc*** infection on non-enzymatic antioxidant content in cabbage AsA-GSH cycle

**Fig. 4:**
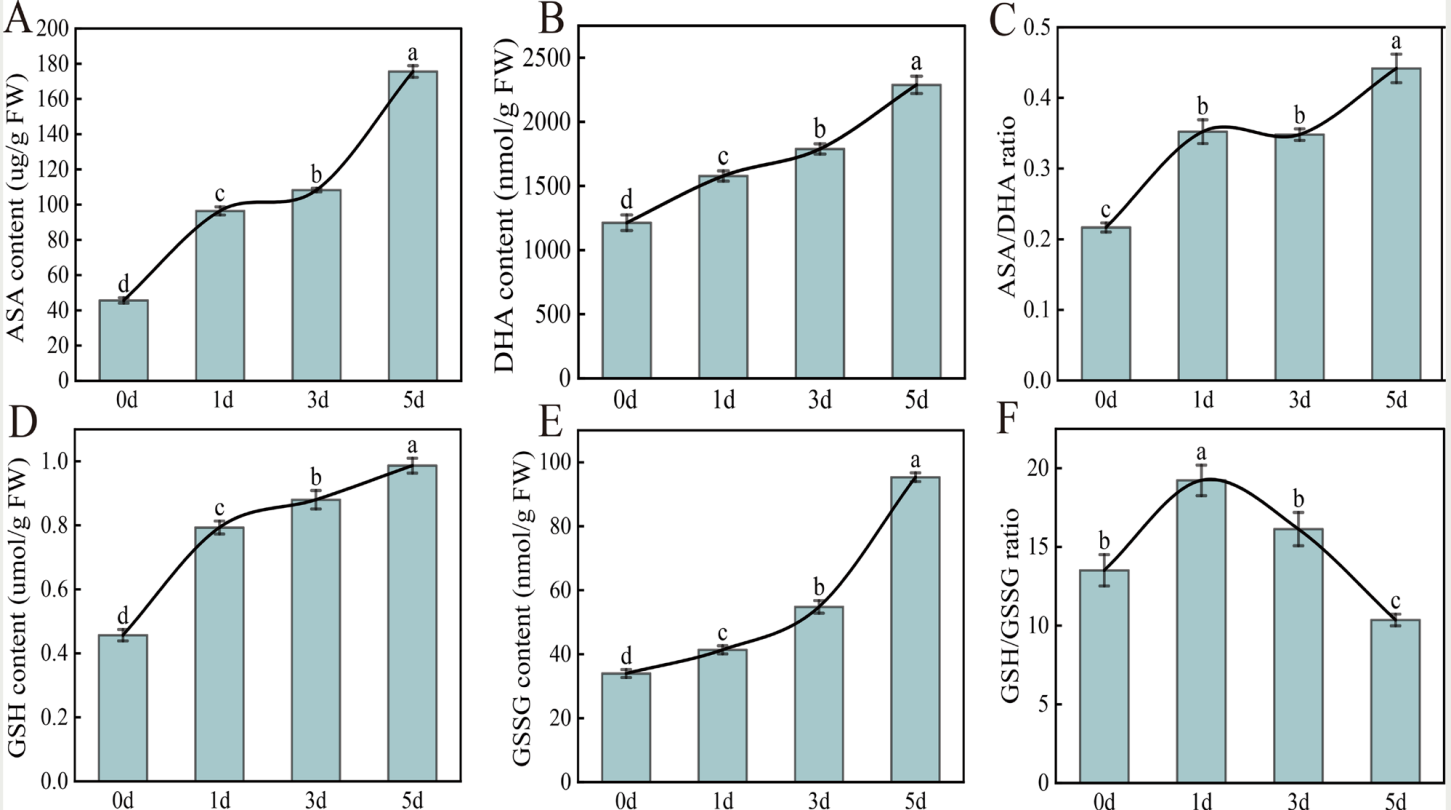
Effect of early *Xcc* infection on non-enzymatic antioxidant contentin AsA-GSH cycle. (**A**) AsA content; (**B**) DHA content; (**C**) AsA/DHA ratio; (**D**) GSH content; (**E**) GSSG content; (**F**)GSH/GSSG ratio

Similar to the changes in the activities of the three antioxidant enzymes, the contents of the non-enzymatic antioxidants AsA, DHA, GSH, and GSSG in the ASA-GSH cycle increased after *Xcc* infection, and the content was highest at 5 d after infection (Fig. 4). Compared to 0 d, the AsA, DHA, GSH, and GSSG contents after 5 d of infection increased by 284.7%, 116.1%, 88.6% and 180.7%, respectively. Among the four non-enzymatic antioxidant substances, the AsA, DHA, and GSH contents showed the same trends and increased sharply in 0–1 d, slowly in 1–3 d, and sharply again in 3–5 d (Fig. 4A, B, D). However, the GSSG content increased slowly from 0 to 3 d and rapidly increased in 3–5 d (Fig. 4E). Because DHA is a reversible oxidizing form of AsA, a change in the AsA/DHA ratio is important in the plant’s environmental stress response. Therefore, we calculated the AsA/DHA ratio, and the results showed that the AsA/DHA ratio increased sharply on 0–1 d, decreased on 1–3 d, and increased sharply on 3–5 d (Fig. 4C). Compared with 0 d, the AsA/DHA ratio increased 1.04 times on 5 d. In addition to the AsA/DHA ratio, the ratio of reduced GSH to oxidized GSSG can be used to assess the redox and detoxification status of the tissue and the protective effect of GSH against oxidative- and radical-mediated cell damage. Based on the measurement results of GSH and GSSG, we also calculated the ratio of GSH to GSSG, and the results showed that the GSH/GSSG ratio increased significantly at 0–1 d and then decreased significantly, with the lowest ratio at 5 d (Fig. 4F). Compared with that at 0 d, the GSH/GSSG ratio decreased by 85.6% on 5 d.

#### Effect of *Xcc* infection on the activity of antioxidant enzymes in cabbage AsA-GSH cycle

**Fig. 5:**
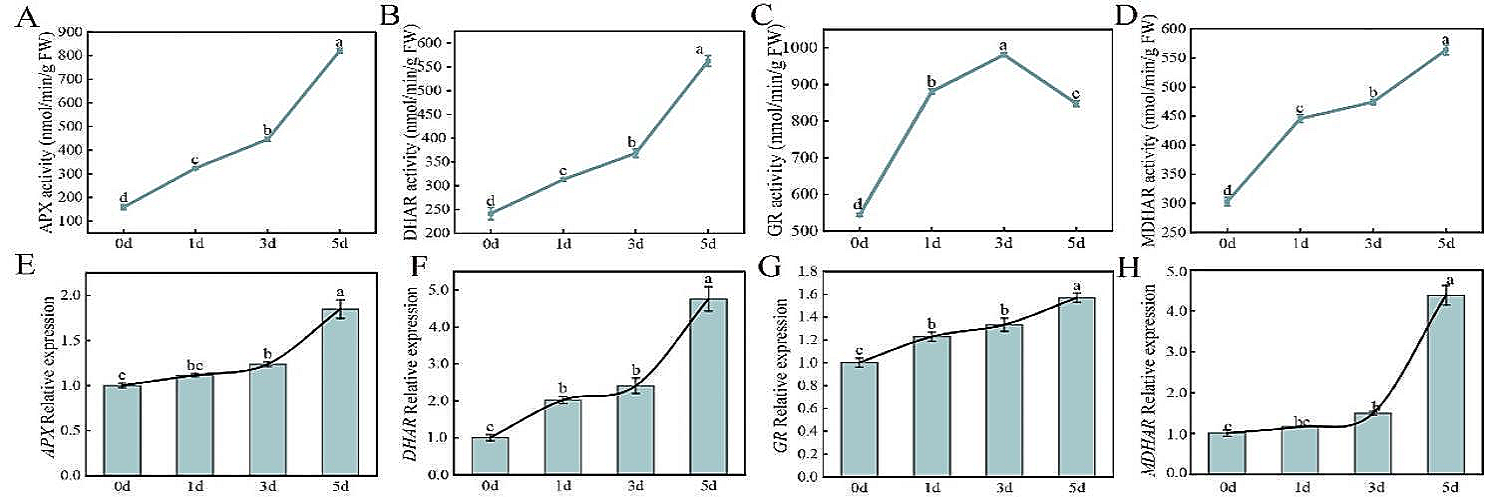
Effect of early *Xcc* infection on antioxidant enzymes activities and related gene expression levels in AsA-GSH cycle.(**A**) APX activity; (**B**) DHAR activity; (**C**) GR activity; (**D**) MDHAR activity; (**E**)*APX* gene expression level; (**F**) *DHAR* gene expression level; (**G**) *GR* gene expression level; (**F**) *MDHAR* gene expression level

Similar to the non-enzymatic antioxidant content trends, the activities of APX, DHAR, GR, and MDHAR antioxidant enzymes in the AsA-GSH cycle showed a general upward trend (Fig. 5). Among the four antioxidant enzymes measured, the activity of APX and DHAR increased slowly and sharply on 3–5 d (Fig. 5A, B), while the activity of MDHAR increased sharply on 0–1 d, slowly increased on 1–3 d, and sharply increased on 3–5 d (Fig. 5D); However, the GR activity increased from 0 to 3 d, reached a maximum at 3 d, and then decreased (Fig. 5C). Compared to 0 d, the activities of APX, DHAR, GR, and MDHAR on 5 d increased 4.18, 1.32, 0.56, and 0.86 times, respectively. Similar to the response trends of related enzyme activities, the expression levels of *APX*, *DHAR*, *GR*, and *MDHAR* also gradually increased, and the increasing trends of *APX*, *DHAR*, and *MDHAR* expression levels were similar to those of the APX, DHAR, and MDHAR enzyme activities (Fig. 5E-H).

### Functional annotation of antioxidation-related differentially expressed genes

**Fig. 6:**
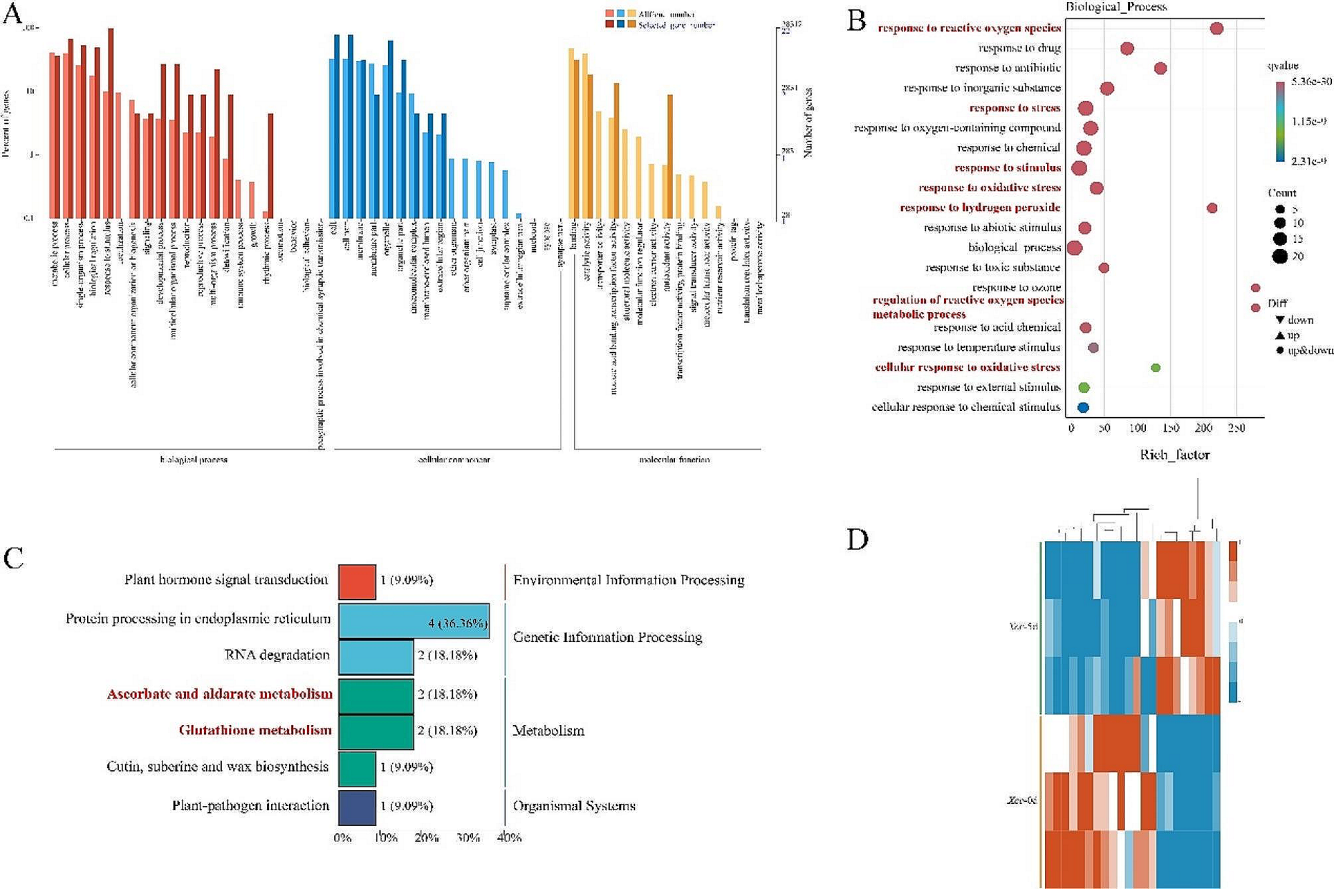
Differentially expressed antioxidant-related genes analysis. (**A**) Gene ontology (GO) classification of differentially expressed reactive oxygen species (ROS) genes. The horizontal coordinate is GO classification, the left side of the vertical coordinate is the percentage of the number of genes, and the right side is the number of genes. (**B**) Gene ontology of ROS genes. The horizontal coordinate is the proportion of the genes we’re interested and all the differentially expressed genes, while the vertical coordinate is each GO annotation entry. The size of the dots represents the number of differentially expressed genes annotated in the pathway, and the color of the dots represents the q-value of the hypergeometric test. (**C**) Kyoto Encyclopedia of Genes and Genomes (KEGG) classification of differentially expressed antioxidant-related genes. The horizontal coordinate is the number of genes annotated to this pathway and its proportion to the total number of genes annotated. (**D**) Cluster heat maps of antioxidant-related genes 0 d and 5 d after *Xcc* infection. Red and blue colour indicate up-regulated and down-regulated genes, respectively. The same below

To explore the molecular mechanism of the complete cabbage genome gene response to *Xcc* stress, the DEGs before and after the *Xcc* infection were analyzed. Gene ontology and KEGG databases were used to explore the potential functions of all antioxidation-related DEGs under *Xcc* infection. Through GO classification, 28,312 DEGs were enriched in 54 pathways, among which cell (7 genes), cell part (7 genes), and response to stimulus (22 genes) had the largest number of differentially enriched genes (Fig. 6A). After *Xcc* infection, the differentially enriched genes related to ROS were enriched in 20 GO terms related to biological processes, and the differentially enriched genes included both up-regulated and down-regulated genes in each term (Fig. 6B). KEGG annotation and functional analysis were performed on differential genes associated with ROS, and it was found that differential genes were mainly involved in “Protein processing in endoplasmic reticulum”, “Glutathione metabolism”, “RNA degradation”, and “Ascorbate and aldarate metabolism” (Fig. 6C). The 22 differentially expressed genes related to ROS were clustered, and the expression levels of the most differentially expressed genes were down-regulated with stress duration (Fig. 6D). Combined with the previous physiological indexes measurement results, we inferred that after *Xcc* infection, the antioxidant capacity of cabbage was affected by “Ascorbate and aldarate metabolism” and “Glutathione metabolism”. Considering that “Ascorbate and aldarate metabolism” and “Glutathione metabolism” play important roles in plant antioxidant stress, we analyzed differential genes related to “Glutathione metabolism” and “Ascorbate and aldarate metabolism” before and after *Xcc* infection.

**Fig. 7:**
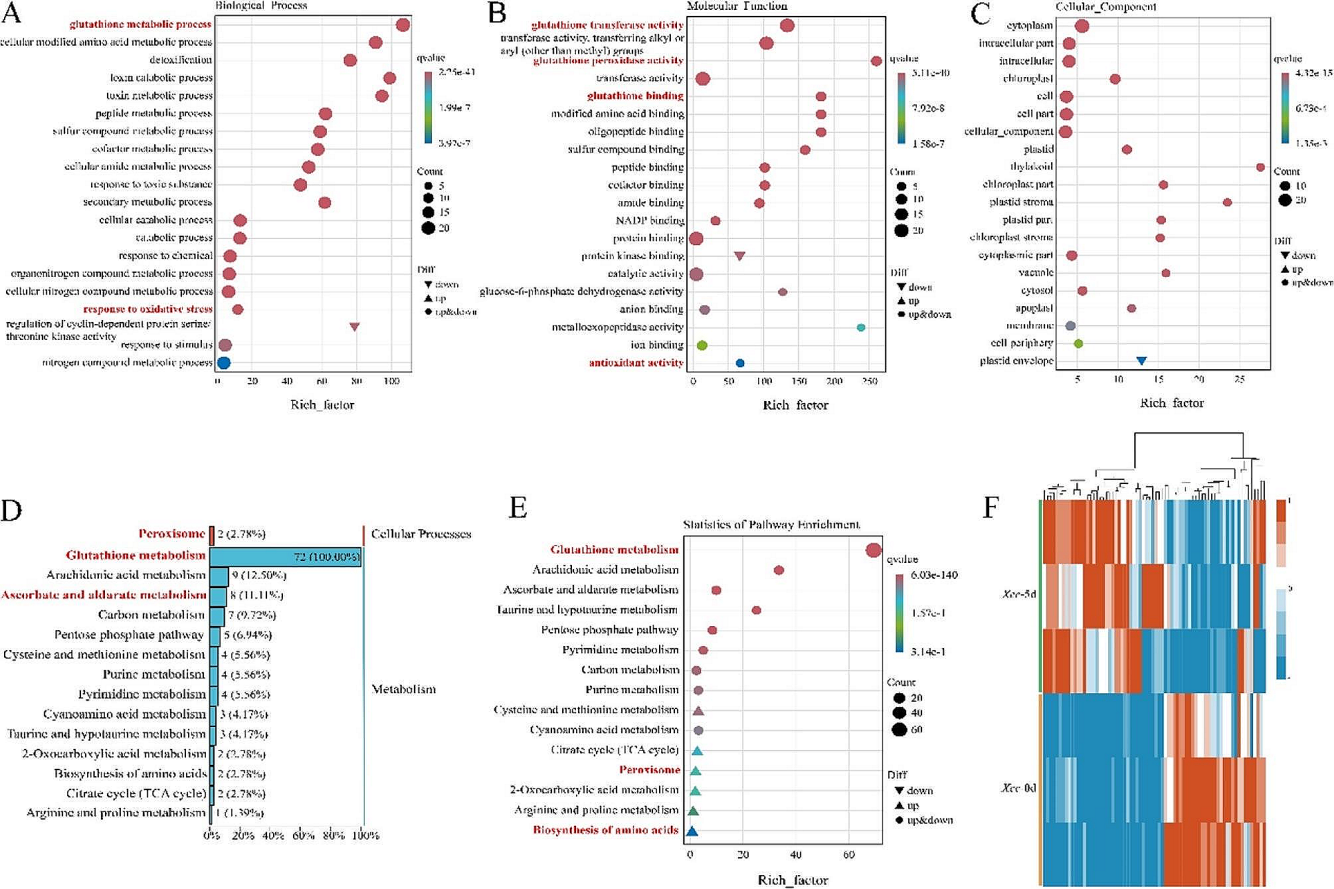
Analysis of differentially expressed glutathione metabolism-related genes. (**A**, **B**, **C**) Gene ontology (GO) classification of differentially glutathione metabolism-related genes. (**D**, **E**) KEGG classification diagram and KEGG bubble diagram of glutathione metabolism. (**F**) Clustering heat map of glutathione metabolism-related differential gene expression

Enrichment analysis of the 72 differential genes related to glutathione metabolism revealed the top 20 significantly enriched pathways in each branch. It was found that in each path, “glutathione metabolic process”, “response to oxidative stress”, “glutathione transferase activity”, “glutathione peroxidase activity”, “glutathione binding,” and “antioxidant activity” all responded to *Xcc* stress (Fig. 7A-C). Differentially expressed genes involved in glutathione metabolism under *Xcc* stress were classified using KEGG. We found that 72, 9, and 8 differential genes were mainly involved in glutathione, arachidonic acid, ascorbate, and aldarate metabolism, respectively (Fig. 7D, E). Next, the expression levels of 72 Glutathione metabolism-related differential genes were screened for cluster analysis. The results showed that 39 genes were up-regulated and 33 genes were down-regulated, respectively (Fig. 7F). Combined with the physiological indexes measured above, we conclude that after *Xcc* infection, cabbage can exert *Xcc* defense capability by enhancing glutathione metabolism.

**Fig. 8:**
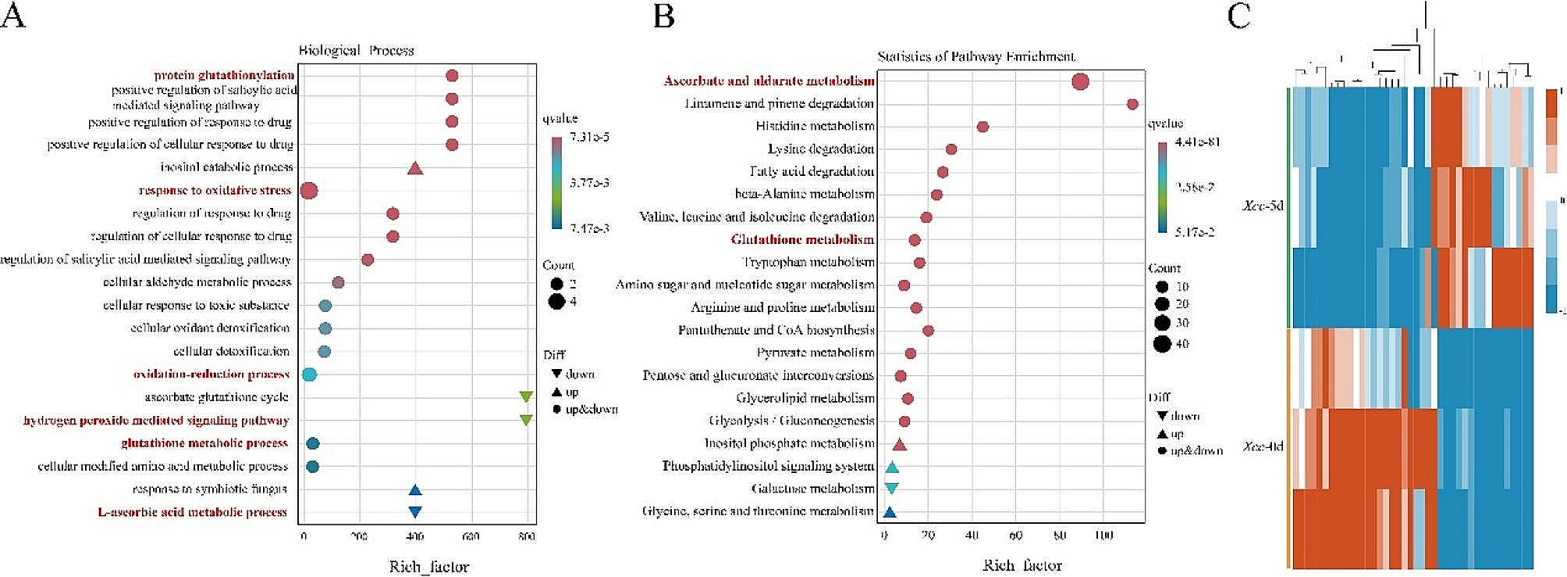
Analysis of differentially expressed ascorbate and aldarate metabolism-related genes. (**A**) GO classification of differentially expressed ascorbate and aldarate metabolism-related genes (biological processes). (**B**) KEGG classification diagram and KEGG bubble diagram of ascorbate and aldarate metabolism. (**C**) Clustering heat map of ascorbate and aldarate metabolism-related differential gene expression

GO enrichment analysis was performed on the 40 differentially expressed ascorbate and aldarate metabolism-related genes, which revealed the top 20 significant pathways in the branches of biological processes. The results showed that “protein glutathionylation”, “response to oxidative stress”, “oxidation-reduction process”, “hydrogen peroxide mediated signaling pathway”, “glutathione metabolic process”, and “L-ascorbic acid metabolic process” are related to cabbage response to *Xcc* infection (Fig. 8A). By classifying the KEGG metabolic pathways of the related differentially expressed genes, we found that glutathione metabolism was also an enriched pathway (Fig. 8B). The expression levels of these 40 differential genes were analyzed using cluster analysis. We found that 24 genes were down-regulated, and 16 genes were up-regulated in response to *Xcc* stress (Fig. 8C). This also indirectly suggests that *Xcc* stress affects the resistance of cabbage by affecting ascorbate and aldarate metabolism.

**Fig. 9:**
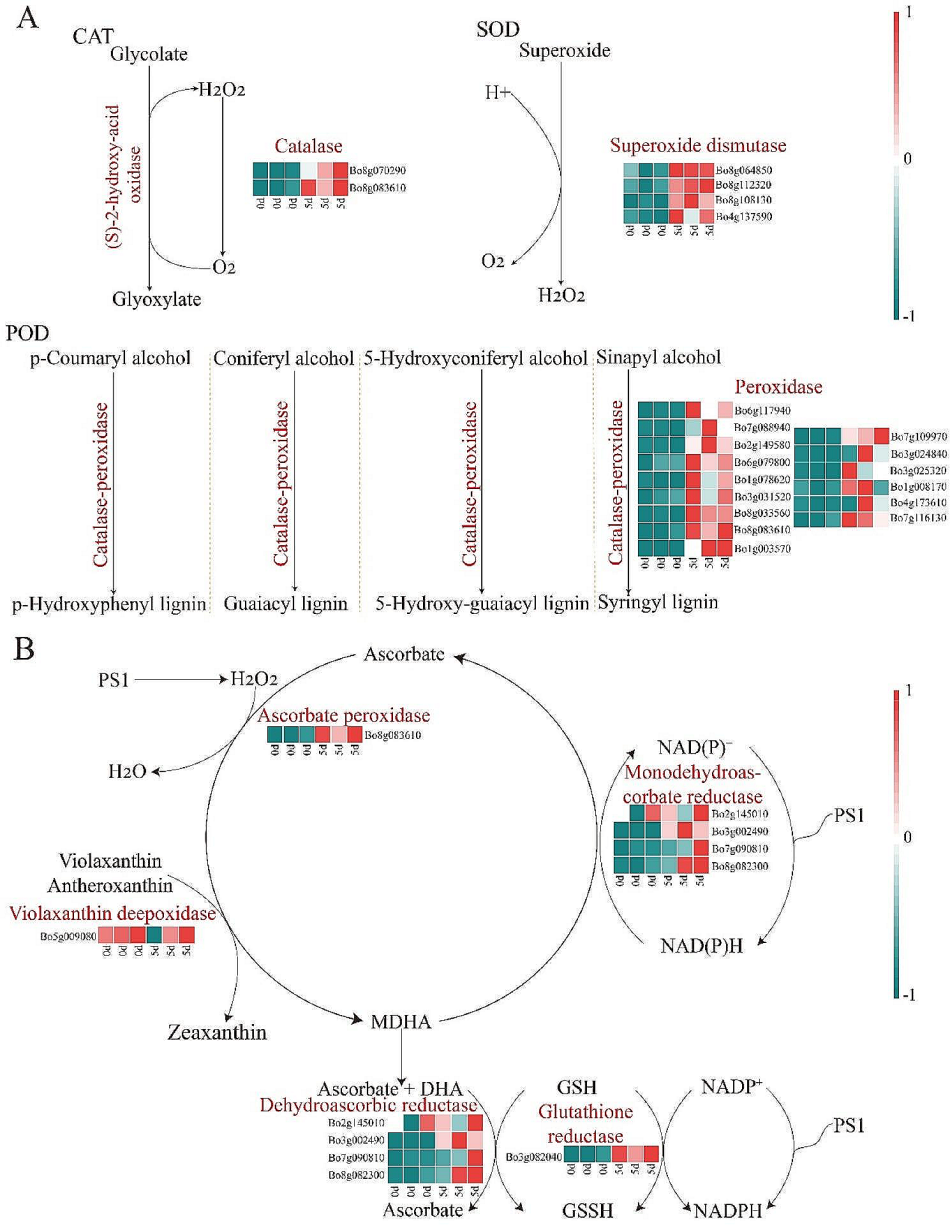
Changes of enzyme activities and related gene expression levels involved in antioxidant defense under different *Xcc* infected days. (**A**) Expression levels of genes related to SOD, POD, and CAT enzymes biosynthesis. (**B**) Expression levels of genes related to APX, DHR, GR, MDHAR, and violanthine decycloxygenase (VDE) enzymes biosynthesis in AsA-GSH

The antioxidant enzymes SOD, POD, and CAT, non-obligate ascorbic acid, and glutathione significantly responded to *Xcc*. The expression levels of genes related to SOD, CAT, POD, and AsA-GSH in cabbage biosynthesis were analyzed after *Xcc* infection. The results showed that the expression levels of most genes related to CAT were significantly up-regulated, while the expression levels of most genes related to SOD and POD were significantly down-regulated (Fig. 9A). Moreover, the expression levels of APX-, DHR-, GR-, MDHAR-, and violanthine decycloxygenase (VDE)-related genes in AsA-GSH showed that the expression levels of APX- and VDE-related genes were down-regulated, one GR related gene was up-regulated, and one GR-related gene was down-regulated, while DHR- and MDHR-related genes were up-regulated (Fig. 9B). Combined with the physiology measured above, we found that the transcriptome data were consistent with the measured physiological indexes.

**Fig. 10:**
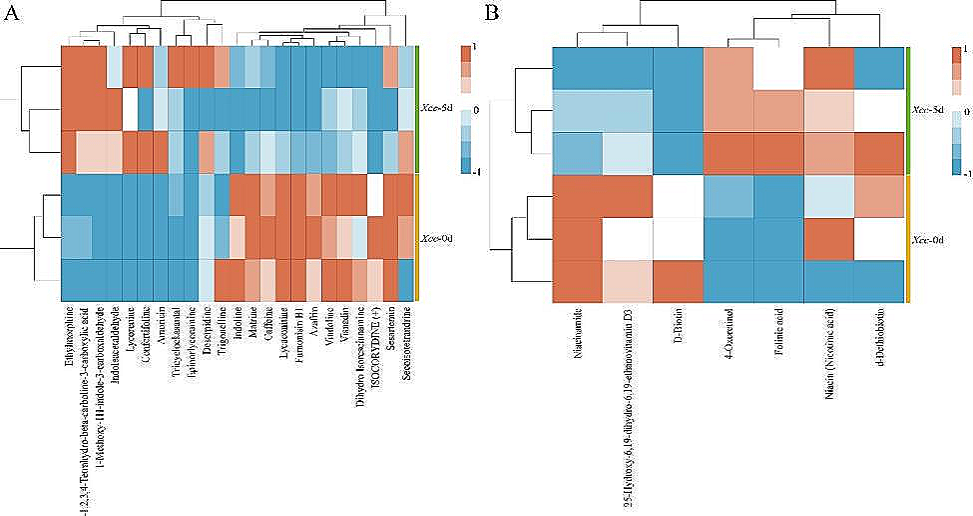
Analysis of differential metabolites in cabbage leaves before and after *Xcc* infection. (**A**) Changes of alkaloid contents. (**B**) Changes of vitamin contents

Antioxidants play important roles in plant disease and pest control; therefore, metabolomic analysis of the antioxidant substances changes in cabbage leaves before and after *Xcc* infection can reveal potential anti-*Xcc* substances. The metabolites in cabbage leaves before and after *Xcc* infection showed significant changes in alkaloid and vitamin contents. The 23 alkaloid contents were normalized and compared. The results showed that the contents of 10 alkaloids (ethylmorphine, L-1,2,3,4-tetrahydro-beta-carboline-3-carboxylic acid, 1-methoxy-1 H-indole-3-carboxaldehyde, indoleacetaldehyde, lycaconitine, confertifoline, amoricin, tricycloekasantal, epinorlycoramine, and deserpidine) increased after *Xcc* infection, while the contents of the another 13 (trigonelline, indoline, matrine, caffeine, lycorenine, fumonisin B1, azafrin, vindoline, visnadin, dihydro isorescinnamine, isocorydine (+), sesartemin, and secoisotetrandrine) decreased with the infection time (Fig. 10A). By analyzing the contents of seven vitamin compounds, we found that the contents of niacinamide, 25-hydroxy-6,19-dihydro-6,19-ethanovitamin D3, and d-biotin decreased, while the content of 4-oxoretinol, folinic acid, niacin, and d-dibiotin increased over time. Changes in the contents of these substances may be related to their participation in black rot resistance (Fig. 10B).

## Discussion

*Xcc* seriously affects the production of cabbage, so it is urgent to study resistance mechanism to prevent and control *Xcc*. Under normal plant growth, the antioxidant system keeps the production and removal of ROS in a state of dynamic balance, the balance is broken under stress. Therefore, it is of great significance to study the cabbage antioxidant mechanism response after *Xcc* infection. Compared with previous studies maily based on the single physiological indexes determination or omics analysis [35–41], the osmoregulatory substances contents, membrane permeability, defense related enzymes activities and AsA-GSH cycle system were studied based on the physiological, transcriptomic and metabolomic levels in our study. Therefore, our study systematically and comprehensively studied the response mechanism of cabbage antioxidant system after inoculation with *Xcc*.

### Changes of osmoregulatory substance content and membrane permeability

Pathogenic bacterial infection can result in abnormal plant growth and development. In phase of pathogen stress, one of the main coping mechanisms of plants is to relieve cell damage by regulating osmotic substances and accumulating them for self-protection [49, 50]. Therefore, it is important to determine the osmotic substances before and after *Xcc* infection to study its infection mechanism of *Xcc*. As plant osmoregulators, soluble sugars, soluble proteins, and free proline (Pro) can accumulate in plants when they are subjected to abiotic stress, play roles in osmotic protection and protein structure stabilization, effectively maintain plant osmotic homeostasis, and play protective roles in plants under adverse conditions such as biological stress [51–53]. Therefore, cell damage and metabolic imbalance were reduced. Similar to the results of other plant diseases, soluble sugar, soluble protein, and Pro contents continued to increase after *Xcc* infection, indicated that cabbage activated defense mechanism to resist osmotic stress caused by *Xcc* [54–56]. Moreover, pathogen infection can increase the relative conductivity and MDA content in plants, resulting in damage to the membrane system and increased membrane permeability [57]. In our study, the MDA content and relative conductivity increased significantly, resulting in damage to the membrane system. Owing to the relative electrical conductivity and MDA levels were negatively correlated with the stability of the cell membrane structure, we concluded that the membrane system was severely damaged after *Xcc* infection.

### Role of ROS and response of antioxidant defense system

*Xcc* and other pathogen infection usually activate the plant ROS production system [27–29]. The increase in ROS not only has a direct toxic effect on pathogens, but can also induce the biosynthesis of phytoprotectin and act as a signal molecule to activate and regulate the expression of related genes in plants [58]. Using two cabbage lines, 40 genes releate to ROS scavenging were identified at the early stages of *Xcc* infection, indicating that ROS plays important roles in the *Xcc* infection [39]. However, excessive ROS can damage plants, trigger and intensify membrane lipid peroxidation and destroy membrane structure and function, eventually leading to accumulation, electrolyte leakage, increased conductivity, and tissue cell death [59, 60]. Therefore, the capacity for plant resistance is not only related to the active oxygen production system, but also to the activity of antioxidant protective enzymes [61, 62]. After *Xcc* infection, the production rate of ROS in cabbage leaf tissue cells increases, and O_2_•^−^ acts as a starting factor to stimulate membrane lipid peroxidation and leads to changes in membrane components to form peroxide products, which in turn increase isoactivity, forming secondary biomass, such as alkaloids and vitamins. This inhibits the propagation of pathogenic *Xcc* and destroys the tissue cells. At the same time, the dynamic balance of O_2_•^−^ is regulated by cellular and internal SOD, POD, and CAT defense enzymes, and the activity of cabbage is rapidly enhanced after *Xcc* infection (O_2_•^−^ is converted to H_2_O_2_ by SOD, then H_2_O_2_ is decomposed into water and oxygen by CAT and POD), which is conducive to the removal of O_2_•^−^ and plays a role in protecting cells, which may be related to the oxidative stress response of the plants themselves [63, 64]. Similar to our findings, the previou study also found the H_2_O_2_ and O_2_•^−^ contents continue to accumulate after *Xcc* infection [36]. Moreover, the SOD and POD activities and the expression levels of related genes and proteins increased after *Xcc* infection, and compared with susceptible material, the increase of enzyme activity and gene expression was more significant [36, 40, 65].

### AsA-GSH cycle system response

Plants can not only remove excess ROS through the antioxidant system, but also maintain the stability of cellular REDOX through the non-enzymatic system, i.e., AsA and GSH as non-enzymatic antioxidants, that directly or indirectly eliminate ROS by participating in the ASA-GSH cycle [31–33]. In this study, by physiological index determination and transcriptomic analysis, we found that AsA and GSH content increased under *Xcc* infection, which may be related to the increased activity of APX and other enzymes in the antioxidant system to reduce the oxidative damage caused by *Xcc* infection. Sufficient GSH needs to be consumed in the ASA-GSH cycle to participate in the REDOX reaction, increase the activity of antioxidant enzymes, and resist the infection of *Xcc*.

### Changes of the disease-resistant substances contents

In addition to the aforementioned antioxidant mechanisms, antioxidant substances are the most important functional components, and their content can be used as an indicator of disease resistance in cabbage [66]. Alkaloids are compounds with a negative oxidation state distributed in most higher plants and are effective antioxidants in living organisms that can react directly with oxygen free radicals [67]. Relevant studies have shown that alkaloids are related to plant disease resistance [68, 69]. In our study, the contents of 10 alkaloids, including Ethylmorphine, L-1,2,3,4-Tetrahydro-beta-carboline-3-carboxylic acid, increased after *Xcc* invasion. Similar with our studies, by investigated the metabolic profile of cabbage leaves during *Xcc* infection, previous study also reported the contents of Isoquinoline N-oxide and other susbstances which belong to alkaloids also dynamic changed [37]. Moreover, vitamin substances in cabbage showed an obvious response after *Xcc* infection, which is consistent with previous reports [70, 71], changes of vitamin contents may be related to the biosynthesis of disease-resistant substances in plants.

Combining physiological and omics data, we conclude that *Xcc* can disrupt the balance of the ROS metabolism system in cabbage, resulting in an increase in free radical generation, which leads to the destruction of the biofilm system and metabolic disorders. Accumulation of ROS in cabbage leaves can be cleared by various pathways, such as increase the antioxidant related enzymes activities, accelerate the AsA-GSH cycle and enhance the biosynthesis of non-specific antioxidant substances. Although there are similarities and differences between our studies and those previously reported, our results provide supporting data and a theoretical basis for subsequent research on the resistance of cruciferous vegetables to black rot.

## Data Availability

The datasets generated during and/or analyzed during the current study are available from the corresponding author on reasonable request.
